# Selective elimination of tumorigenic cells from mixed culture of normal and tumorigenic cells using hybrid liposomes aimed at realizing of cell therapy

**DOI:** 10.1007/s10616-023-00613-y

**Published:** 2024-02-13

**Authors:** Riko Jinno, Moe Tanaka, Yuji Komizu, Yoko Matsumoto, Taku Matsushita, Seiichi Ishida

**Affiliations:** Division of Applied Life Science, Graduate School of Engineering, Sojo University, Kumamoto, Japan

**Keywords:** Regenerative medicine, Tumorigenic cells, Safety, Hybrid liposomes, Selective elimination

## Abstract

While induced pluripotent stem (iPS) cells are expected to be a cell source for regenerative medicine, they also have tumorigenic properties owing to their proliferative potential. During the manufacturing of regenerative medicine products, undifferentiated iPS cells and malignant transformed cells may be mixed in the cell culture population. Therefore, it is essential to eliminate tumorigenic cells selectively. In this study, a mixed culture of normal human fetal hepatocytes (Hc cells) and human hepatocellular carcinoma cells (HuH-7 cells) was used as a cell population model to be used as regenerative medicine products, and the selective elimination of HuH-7 cells by hybrid liposomes (HL) was analyzed. HL tended to fuse and accumulate more in HuH-7 cells due to larger fluidity of plasma membrane for HuH-7 cells than that for Hc cells. In a mixed culture of Hc and HuH-7 cells, HL selectively eliminated HuH-7 cells while allowing Hc cells to remain viable. In addition, HL treatment for the mixed culture of Hc and HuH-7 cells suppressed the tumorigenicity of HuH-7 cells. Therefore, HL selectively fused and accumulated in tumorigenic cells in a mixed cell culture of normal and tumorigenic cells, and eliminated tumorigenic cells while allowing normal cells to remain viable. The results of this study suggest the potential of HL in eliminating tumorigenic cells during the manufacturing of regenerative medicine products. Thus, HL could be expected to contribute to the development of safe regenerative medical products.

**Supplementary Information:**

The online version contains supplementary material available at 10.1007/s10616-023-00613-y.

## Introduction

Pluripotent stem cells, such as embryonic stem cells (ES cells) and induced pluripotent stem cells (iPS cells), can differentiate into all three embryonic-like cell types. Therefore, pluripotent stem cells have attracted attention as a cell source for cell therapy for various diseases and injuries (Yamanaka 2020). Among pluripotent stem cells, iPS cells exhibit properties similar to those of ES cells in terms of karyotype, phenotype, telomerase activity, and differentiation potential (Volarevic et al. 2018). In addition, because iPS cells have proliferative potential, they are easy to handle as research materials, and have been used in regenerative medicine research. iPS cells can be cultured in large quantities, making it possible to manufacture regenerative medical products. Therefore, regenerative medicine using iPS cells is expected to solve problems, such as the persistent issue of a shortage of organ donors for transplantation therapy (Rouchi and Mahdavi-Mazdeh 2015).

Currently, in the manufacturing of regenerative medical products, iPS cells are cultured on a large scale to achieve the number of cells required for transplantation. iPS cells are then induced to differentiate into target tissue cells. However, their high proliferative capacity enables the formation of tumorigenic cell populations, and their safety must be considered after transplantation (Ahrlund-Richter et al. 2008; Kuroda et al. 2013; Volarevic et al. 2018; Yamanaka 2020). In addition, various environmental stresses and incomplete differentiation may occur during the manufacture of regenerative medicine products owing to mass culture. This may cause lead to the presence of undifferentiated iPS cells or malignant transformed cells in the final cell population (Szablowska-Gadomska et al. 2011). Therefore, to create safe regenerative medicine products, it is essential to reduce tumorigenic cells by improving mass culture process and differentiation induction technologies and by selectively eliminating of tumorigenic cells.

We have been investigating the anticancer effects of hybrid liposomes (HL) as a cancer treatment. HL can be easily prepared by sonication of vesicular molecules such as phospholipids and micellar surfactants in buffer solutions, and are biocompatible medical material that is completely free of organic solvents during preparation (Ueoka et al. 1985, 1988). HL, composed of 90 mol% L-α-dimyristoylphosphatidylcholine (DMPC) and 10 mol% polyoxyethylene(n)dodecyl ethers (C_12_(EO)_n_), have a stable hydrodynamic diameter with a narrow size distribution range at 37℃ (Nakano et al. 2002). Interestingly, the ratio of the component lipids of plasma membranes differs significantly between normal and cancer cells (Marique and Hildebrand 1973; Rethy et al. 1975). In our previous reports, membrane fluidity for cancer cells were different from that for normal cells due to lipid component of cell membrane. HL recognized this fluidity and selectively fused and accumulated in cancer cells with high membrane fluidity (Yukihara et al. 2010; Komizu et al. 2011a, b). Furthermore, remarkable inhibitory effects of HL on the growth of various cancer cells have been obtained while causing in vitro apoptosis (Imamura et al. 1996, 1997; Matsumoto et al. 1999a, b; Matsumoto et al. 1999a, b), in vivo animal models (Ichihara et al. 2003, 2011, 2012, 2014). Regarding evaluation of safety, HL caused no abnormalities in body weight or hematological and biochemical tests in chronic toxicity studies using normal rats (Ichihara et al. 2003, 2008). Furthermore, HL have been clinically administered intravenously in a total of 10 cases in 8 different cancers, including liver cancer. There have been no abnormal findings based on various hematological and biochemical tests, and the patients have achieved long-term survival of more than one year without side effects (Ichihara et al. 2008). Therefore, HL can be considered a safe reagent for introduction in vivo.

For regenerative medicine products intended for cell transplantation, it is desirable that the safety of reagents for elimination of tumorigenic cells be guaranteed and that they do not adversely affect normal differentiated cells that will become transplanted cells. In this regard, HL are a reagent that is safe even when mixed with living organisms and does not act on normal cells, so they can be expected to serve as a reagent for eliminating tumorigenic cells in regenerative medicine.

This study aimed to selectively eliminate tumorigenic cells mixed in a cell population using HL, mimicking the manufacturing process of regenerative medicine products to create tumorigenic-free regenerative medicine products.

In previous studies, in vitro evaluations of the effects of HL on normal and tumorigenic cells were used to compare the results obtained in each cell culture system (Nakano et al 2002; Inamura et al. 2021). However, the selective elimination of tumorigenic cells by HL in mixed cell cultures has not been investigated. Therefore, in this study, we assessed the ability of HL to selectively eliminate tumorigenic cells mixed with normal cells by mixing normal human fetal hepatocytes (Hc cells) and hepatocarcinoma cells (HuH-7 cells).

Hc and HuH-7 cells were cultured, and the growth-inhibitory effect of HL on Hc and HuH-7 cells was examined. Then, the selective elimination of tumorigenic cells by HL in mixed cultures of Hc and HuH-7 cells was evaluated by comparison with doxorubicin (DOX), a typical anticancer drug, as a reference.

## Materials and methods

### Cells and medium

Normal human fetal hepatocytes (Hc cells) were obtained from DS Pharma Biomedical Co. (Osaka, Japan) with a certification of informed consent for research and were cultured in CS-C medium (DS Pharma Biomedical Co.). Human hepatocellular carcinoma cells (HuH-7 cells) were purchased from RIKEN CELL BANK (Ibaraki, Japan) and incubated in Dulbecco’s modified Eagle’s medium (DMEM) (FUJIFILM Wako Chemicals, Osaka, Japan) supplemented with 10% fetal bovine serum (FBS; CAPRICORN, Hessen, Germany) and penicillin–streptomycin solution (FUJIFILM Wako Chemicals). In addition, Hc and HuH-7 cells were mixed-inoculated in CS-C medium (DC Pharma Biomedical Co.) such that the percentage of HuH-7 cells to total cells was 0, 25, 50, 75, and 100%. These cells were cultured in a 5% CO_2_ humidified incubator at 37 °C.

### Anti-cancer agent

Doxorubicin hydrochloride (DOX) was purchased from FUJIFILM Wako Chemicals and dissolved in sterile water to a concentration of 10 mM (stock solution) to serve as an anti-cancer reference.

### Preparation of HL

HL were prepared by sonicating a mixture containing 90 mol% L-α-dimyristoyl phosphatidylcholine (DMPC; NOF Co., Tokyo, Japan) and 10 mol% polyoxyethylene (n) dodecyl ether (C_12_(EO)_n_, n = 25) (Nikko Chemicals, Tokyo, Japan) in a 5% glucose solution. For sonication of HL, a bath-type sonicator (ULTRASONIC-CLEANER-WT-200-M, Tokyo, Japan) was used at 45℃ and 300 W, after which HL were filtered using a 0.20 µm cellulose acetate filter (Advantec, Tokyo, Japan).

The size of HL at 25 °C was determined via the dynamic light scattering method (ELS-8000, Otsuka Electronics, Osaka, Japan). The hydrodynamic diameter of the HL was approximately 34 nm with a narrow size distribution range, and the HL remained stable for more than 3 weeks.

### Selection of medium for mixed culture

The medium used for the mixed culture of Hc and HuH-7 cells was evaluated by a cell growth curve using CS-C medium (DS Pharma Biomedical Co.) and DMEM (FUJIFILM Wako Chemicals). The cells were seeded at 1.0 × 10^4^ cells/cm^2^ and counted using an Auto Cell Counter (ADAM-MC, Digital Bio, Tokyo, Japan) after 1, 2, 3, 5, and 7 d of cell culture. The medium was changed once every 2 days.

### Cell membrane fluidity measurement

The membrane fluidity of intact cells was evaluated using the fluorescence depolarization method with the fluorescent probe1,6-diphenyl-1,3,5-hexatriene (DPH; Nacalai Tesque, Kyoto, Japan). The cells were treated with 0.05% trypsin–EDTA and suspended in HBSS (2.0 × 10^4^ cells/ml). DPH was added to the cell suspension at a final concentration of 2 µM and allowed to stand for 15 min at 37℃. The fluorescence polarization (*P*) of DPH was measured using a fluorescence spectrophotometer (F-7100; Hitachi, Tokyo, Japan).

### Accumulation of HL in the cell membrane

HL/NBDPC was prepared by sonicating a mixture containing 86 mol% DMPC, 10 mol% C_12_(EO)_25,_ and 4 mol% the fluorescent probe 1-palmitoyl-2-{12-[(7-nitro-2-1, 3-benzoxadiazol-4-yl) amino] dodecanoyl}-sn-glycero-3-phos-phocholine (NBDPC; Avanti Polar Lipids, Inc., AL, USA) in a 5% glucose solution, similar to HL. NBDPC liposomes were prepared by sonication of NBDPC alone in 5% glucose solution.

HuH-7 cells were dye-labeled with 5 µM cell tracking solution (CellTracker™ Red CMFDA, Thermo, Massachusetts, USA) for 30 min. Unlabeled Hc and dye-labeled HuH-7 cells were then collected, mixed, and seeded such that the percentage of HuH-7 cells to the total number of cells was 0, 25, 50, 75, and 100%. Mixed Hc and HuH-7 cells after seeding were cultured at 37° C in a 5% CO_2_ incubator for 24 h, following which the cells were treated with HL/NBDPC for 1 h at a final concentration of [DMPC] = 300 µM, [C_12_(EO)_25_] = 33.3 µM, and [NBDPC] = 13.9 µM in the medium. NBDPC liposomes were treated for 1 h at a final concentration of 13.9 µM in the medium.

The fused accumulation of HL/NBDPC or NBDPC in the membranes of Hc and HuH-7 cells was evaluated by confocal laser microscopy (Olympus, IX83-CSU-X1, Tokyo, Japan) using 488 nm excitation light for NBDPC and 561 nm for CellTracker™ Red CMFDA.

### Cell growth inhibition assay

DOX or HL cytostatic assays on Hc and HuH-7 cells were performed using the WST-8 (2-(2-methoxy-4-nitrophenyl)-3-(4-nitrophenyl)-5-(2, 4-disulfophenyl)-2H tetrazolium, monosodium salt) assay (Cell Counting Kit-8, Dojindo, Kumamoto, Japan). Hc or HuH-7 cells were each seeded in a 96-well plate at 5.0 × 10^3^ cells/well and cultured at 37° C in a CO_2_ incubator for 24 h. DOX (0–100 µM) or HL ([DMPC] = 0–3000 µM]) was then added and cells were cultured for an additional 48 h. The WST-8 solution was then added and incubated for 2 h.

The absorbance was measured at 450 nm using a microplate reader (Thermo Fisher Scientific). The inhibitory activity of DOX or HL on Hc or HuH-7 cell proliferation was assessed by calculating A_mean_/A_control_. Here, A_mean_ and A_control_ indicate the absorbance of the water-soluble formazan in the presence and absence of DOX and HL, respectively. Next, the calculated cytostatic rates of DOX and HL were analyzed using GraphPad Prism9, and sigmoid curves and 20%, 50%, and 80% cytostatic concentrations (IC_20_ values, IC_50_ values, IC_80_ values) were calculated.

### Confocal laser microscopy

HuH-7 cells were dye-labeled with 5 µM cell tracking solution (CellTracker™ Green CMFDA (5-chloromethylfluorescein diacetate), Thermo) for 30 min. Unlabeled Hc and dye-labeled HuH-7 cells were collected, mixed, and seeded into 35 mm glass bottom dishes (MatTek, P35GC-0-14-C, Massachusetts, USA) such that the percentage of HuH-7 cells to the total number of cells was 0, 25, 50, 75, and 100%. Mixed Hc and HuH-7 cells after seeding were cultured at 37° C in a 5% CO_2_ incubator for 24 h, treated with DOX (0, 0.1, 1, 10 µM) or HL ([DMPC] = 0, 200, 400, 600 µM), and cultured for 48 h. The cells were then incubated with a nuclear staining solution (TO-PRO™-3 Iodide, Invitrogen) for 15 min and mounted with an antifade agent (SlowFade™ Diamond Antifade Mountant, Invitrogen). They were then observed using a confocal laser scanning microscope (TCS-SP; Leica Microsystems, Wetzlar, Germany) using a 488 nm Ar laser line and a 633 nm He/Ne laser line.

### Flow cytometry analysis

HuH-7 cells were dye-labeled with 5 µM cell tracking solution (CellTracker™ Green CMFDA (5-chloromethylfluorescein diacetate), Thermo, C7025) for 30 min. Unlabeled Hc and dye-labeled HuH-7 cells were collected, mixed, and seeded into 60 mm dish (BD FALCON, Arizona, USA) such that the percentage of HuH-7 cells to the total number of cells was 0, 25, 50, 75, and 100%. Mixed Hc and HuH-7 cells after seeding were cultured at 37° C in a 5% CO_2_ incubator for 24 h, treated with DOX (0, 0.1, 1, 10 µM) or HL ([DMPC] = 0, 200, 400, 600 µM), and cultured for 48 h. Then, the mixed cells were detached with a 0.05% trypsin–EDTA solution, and the cell number was adjusted to 1.0 × 10^6^ cells/sample. Flow cytometry was performed using a CytoFLEX with a 488 nm laser line. Data were analyzed using CytExpert software (Beckman Coulter Inc., California, USA).

### Soft agar colony formation assay

Cell tumorigenicity was assessed using a soft agar colony formation assay. Concentrated CS-C medium containing agar prepared according to DS Pharma Biomedical Co., DMEM/Nutrient Mixture F-12 (Thermo Fisher Scientific, Ashland, NC, USA) containing 10% BSA and 50 ng/mL recombinant human FGF-acidic (PeproTech Inc., Rocky Hill, NJ, USA) was used for the assay.

First, 350 µL of 0.5% agar medium (1% agar solution:2 × DMEM/F-12 = 1:1) was added to a 48-well plate (Sumitomo Bakelite Co., Ltd., Tokyo, Japan) and solidified as the lower-layer agar. Next, 0.5% agar medium and cell suspension were mixed in a ratio of 2:1 to form a semisolid to prepare upper agar; 50 µL of upper agar was layered on top of the lower agar. The cells were seeded at 1000 cells/well and cultured for 9 d at 37℃ in 5% CO_2_ and 95% humidity.

After 9 d of culture, surviving colonies in the top agar were stained with 5 µM Calcein-AM (DojinDo, Kumamoto, Japan). The stained colonies were imaged using a fluorescence microscope (EVOS FL; Thermo Fisher Scientific Inc., Waltham, MA, USA). Colony numbers were analyzed from the photographs using the ImageJ software (National Institutes of Health, Bethesda, Md., USA). A stained colony > 80 µm in diameter, corresponding to > 20 cells, and an area > 5700 µm^2^ were defined as a tumorigenic colony (Meyskens et al. 1984), which is tumorigenic colonies are formed through cell proliferation.

### Statistical processing

The results are presented as mean ± standard deviation (SD). Data were statistically analyzed using Student’s t-test. Differences were considered statistically significant at *p* < 0.05.

## Results

### CS-C medium is suitable for mixed cultures of Hc and HuH-7 cells

**Fig. 1:**
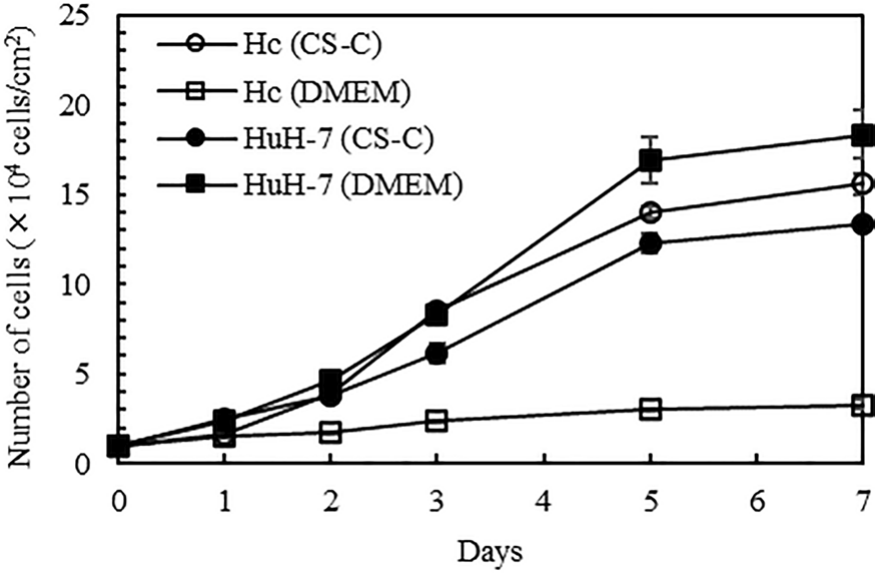
Selection of medium for the mixed culture of Hc and HuH-7 cells. The medium used for the mixed culture of Hc and HuH-7 cells was determined by evaluating cell proliferation capacity over 7 days. Growth of Hc and HuH-7 cells in CS-C medium, the recommended medium for Hc cell culture, and DMEM containing 10% FBS, the recommended medium for HuH-7 cell culture, was evaluated. Data represent the mean ± SE (n = 3)

First, the optimal medium used for the mixed culture of Hc and HuH-7 cells was examined. Two media namely, CS-C medium, which is the recommended medium for Hc cell culture, and DMEM containing 10% FBS, which is the recommended medium for HuH-7 cell culture were evaluated by constructing growth curves for Hc and HuH-7 cells for each medium (Fig. 1).

Hc showed high growth ability in CS-C medium but not in DMEM containing 10% FBS. In contrast, HuH-7 cells showed high proliferative ability in both CS-C medium and DMEM containing 10% FBS. Therefore, the CS-C medium, which showed a high proliferative capacity for both cell types, was suitable for mixed cultures of Hc and HuH-7 cells and all subsequent experiments were performed using the CS-C medium.

### The membrane fluidity of HuH-7 cells is greater than that of Hc

We previously reported that HL detect differences in membrane fluidity between tumorigenic and normal cell membranes and that they fuse and accumulate in tumorigenic cell membranes with high cell membrane fluidity (Komizu et al. 2011a, b). In this study, we investigated the membrane fluidity, fusion and accumulation of HL in Hc cells, which are normal cells, and HuH-7 cells, which are tumorigenic cells.

**Fig. 2:**
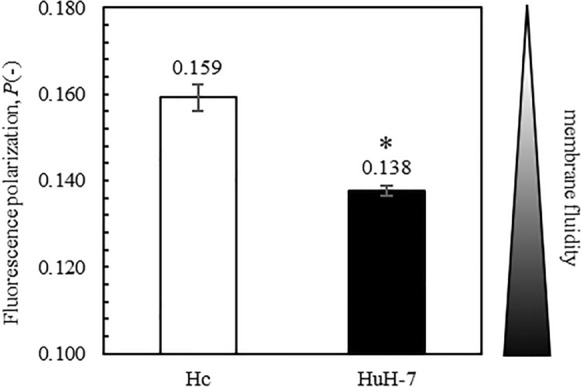
Comparison of cell membrane fluidity between Hc and HuH-7 cells. The cell membrane fluidity of Hc and HuH-7 cells cultured in the CS-C medium was measured. Data represent the mean ± SE (n = 3). *Significant difference compared to Hc (*p* < 0.01)

Membrane fluidity, fusion and accumulation of HL in Hc cells cultured in the CS-C medium have been reported previously (Inamura et al. 2021). However, the type of spectrofluorometer used to measure cell membrane fluidity and the method for evaluating fusion and accumulation of HL differed from those used in previous studies (Inamura et al. 2021). Therefore, we investigated the membrane fluidity, fusion and accumulation of HL in Hc and HuH-7 cells newly cultured in a CS-C medium using the fluorescence depolarization method with a spectrofluorometer (F-7100). The degree of fluorescence polarization (*P* value) of HuH-7 cells was lower than that of Hc cells, indicating that the membrane fluidity of HuH-7 cells was greater than that of Hc cells (Fig. 2).

### HL selectively fuse and accumulate in HuH-7 cells under mixed culture conditions of Hc and HuH-7 cells

**Fig. 3:**
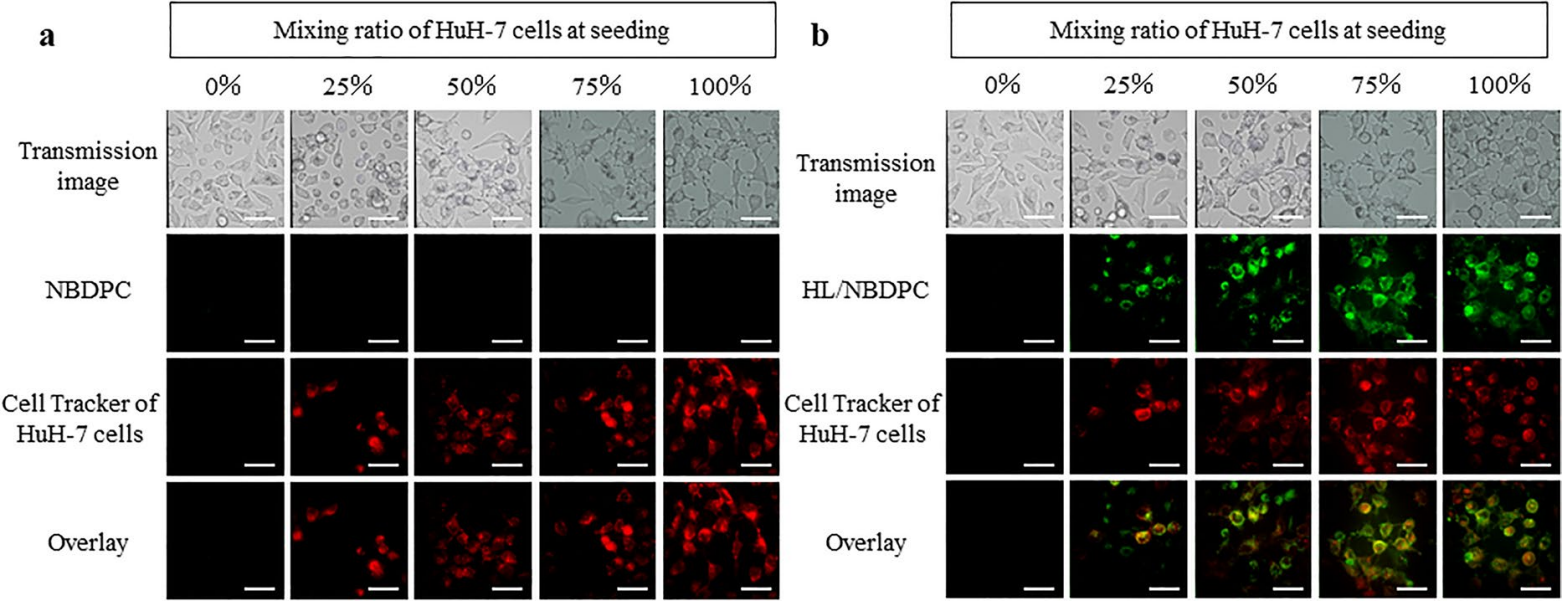
Confocal laser microscope observation of accumulation of 300 µM HL containing fluorescently labeled lipid (NBDPC) in mixed Hc and HuH-7 cells. Confocal laser micrographs of mixed Hc and HuH-7 cells treated with NBDPC liposomes **a** or HL/NBDPC **b** for 1 h. Scale bar represents 50 µm

Next, fusion and accumulation of HL into the membrane of Hc or HuH-7 cells using NBDPC liposomes or HL/NBDPC was evaluated by confocal laser microscopy. Fusion and accumulation of HL/NBDPC was evaluated in the mixed culture (Table 1) to assess the selectivity of HL for HuH-7 cells using HuH-7 cell tracking. HuH-7 cells were tracked by dye-labeling only HuH-7 cells using a Red Cell Tracker (Fig. 3).

**Table 1:**
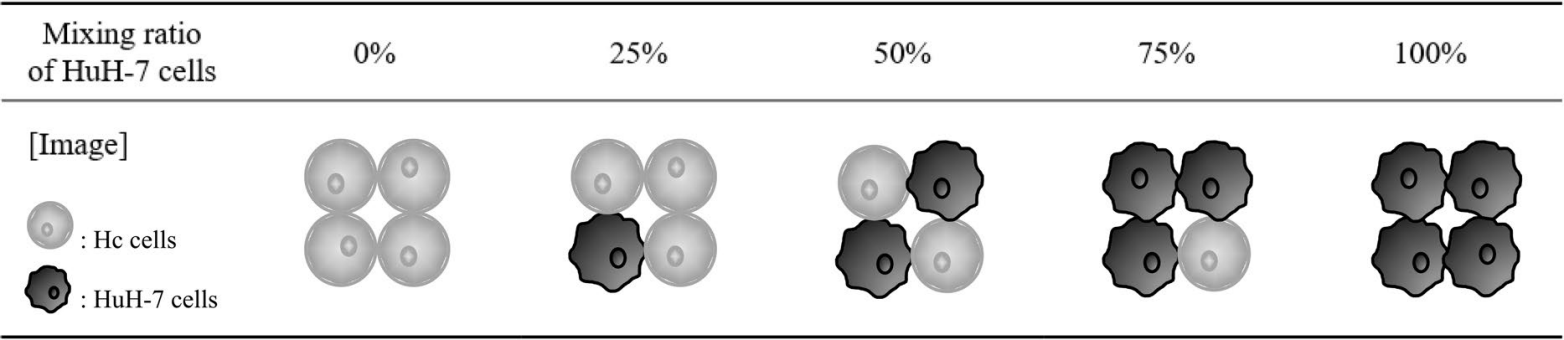
Proportion of Hc and HuH-7 cells in mixed seeding

The fluorescence of NBDPC was not observed in Hc or HuH-7 cells treated with NBDPC liposomes (Fig. 3a). Therefore, NBDPC liposomes did not fuse and accumulate in the cell membrane. HL/NBDPC did not fuse and accumulate under the HuH-7 0% condition, which indicates its interaction with only Hc cells. However small amounts of HL/NBDPCs were observed to fuse and accumulate in Hc cells under 25–75% conditions. Most HL/NBDPCs fused and accumulated in HuH-7 cells, and in 100% HuH-7 cells, HL/NBDPCs fused and accumulated in all cells.

Therefore, HL were more likely to fuse and accumulate in HuH-7 cells than in Hc cells. These results suggest that HL discriminate between normal and tumorigenic cells in the same environment, and selectively fuse and accumulate in tumorigenic cells.

### HL inhibit the growth of Hc and HuH-7 cells in a narrow concentration range and eliminate only HuH-7 cells

**Fig. 4:**
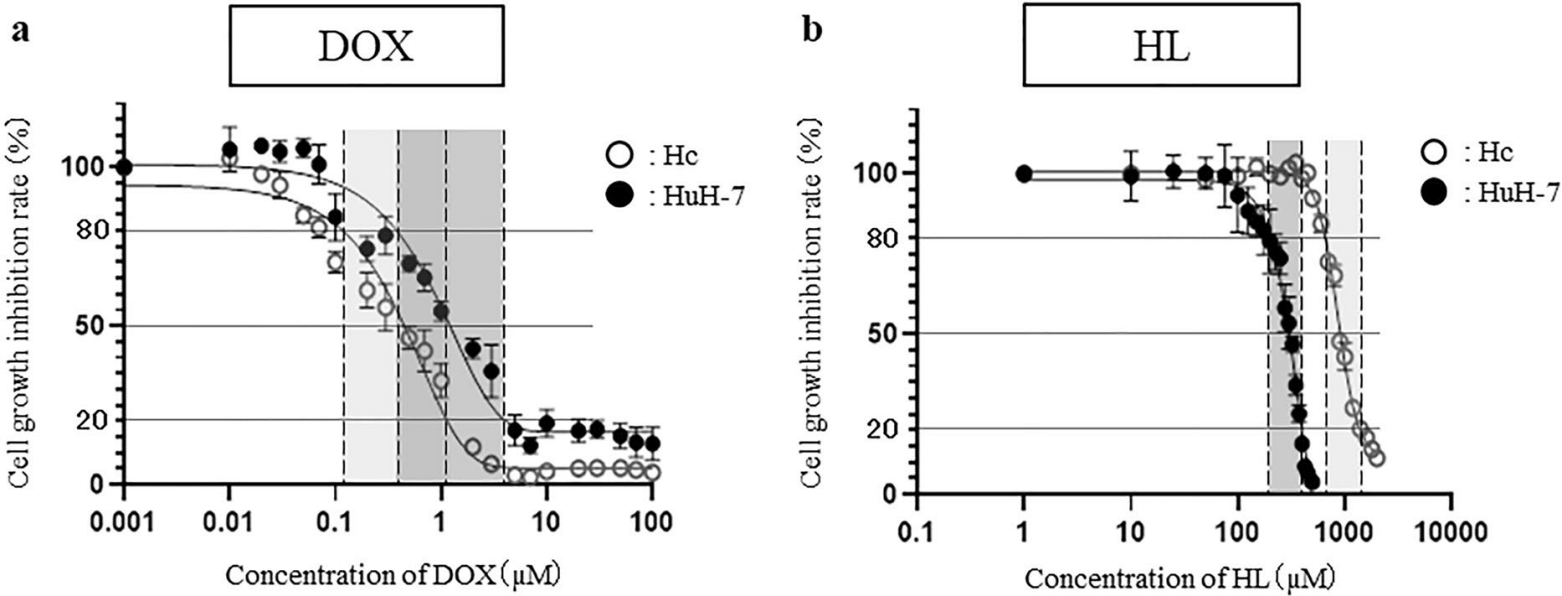
Cell growth inhibition ability of DOX or HL in Hc or HuH-7 cells. Hc or HuH-7 cells were treated with various concentrations of DOX (0.01–100 µM) and HL (1–2000 µM) for 48 h. The working concentration range of DOX (**a**) and HL (**b**) between 20% cytostatic concentration (IC_20_) and 80% cytostatic concentration (IC_80_) for each cell is shown. Data represent the mean ± SE (n = 4)

To evaluate whether HL selectively eliminates HuH-7 cells in a mixed culture of Hc and HuH-7 cells, the effect of HL on Hc and HuH-7 cells was assessed using a cytostatic assay (Fig. 4). The cytostaticity of HL was compared with that of DOX, a representative anticancer drug. The inhibitory concentration of DOX from IC_20_ to IC_80_ ranges from 4.00 × 10^–2^ to 1.91 µM for Hc cells and from 3.16 × 10^–1^ to 9.91 µM for HuH-7 cells (Table 2). The inhibitory concentration range of DOX overlapped between Hc and HuH-7 cells (Fig. 4a). On the other hand, HL had an inhibitory concentration of IC_20_ and IC_80_ ranging from 6.6 × 10^2^ to 1.34 × 10^3^ µM for Hc cells and from 2.00 × 10^2^ to 3.92 × 10^2^ µM for HuH-7 cells (Table 3). The inhibitory concentration range of HL did not overlap between Hc and HuH-7 cells (Fig. 4b). Therefore, HL could be used to selectively eliminate HuH-7 cells under a working concentration ranging from 3.92 × 10^2^ to 6.61 × 10^2^ µM.

**Table 2:** Proliferation of Hc or HuH-7 cells under 20, 50, and 80% inhibitory concentrations (IC_20,_ IC_50_, and IC_80_, respectively) of DOX

	IC_20_ ± S.E. (µM)	IC_50_ ± S.E. (µM)	IC_80_ ± S.E. (µM)	Number of experiments
Hc cell	1.05 × 10^–1^ ± 1.85 × 10^–2^	3.41 × 10^–1^ ± 7.03 × 10^–2^	1.08 ± 1.29 × 10^–1^	4
HuH-7 cell	3.16 × 10^–1^ ± 6.80 × 10^–2^	1.13 ± 2.41 × 10^–1^	9.91 ± 2.77 × 10^–1^	4

**Table 3:** Proliferation of Hc or HuH-7 cells under 20, 50, and 80% inhibitory concentrations (IC_20,_ IC_50_, and IC_80_, respectively) of HL

	IC_20_ ± S.E. (µM)	IC_50_ ± S.E. (µM)	IC_80_ ± S.E. (µM)	Number of experiments
Hc cell	6.61 × 10^2^ ± 5.10	9.14 × 10^2^ ± 6.98	1.40 × 10^3^ ± 2.24 × 10^1^	4
HuH-7 cell	2.00 × 10^2^ ± 1.62	3.05 × 10^2^ ± 4.74	3.92 × 10^2^ ± 4.26	4

### HL selectively eliminated HuH-7 cells without altering the viability of Hc cells in mixed cultures

**Fig. 5:**
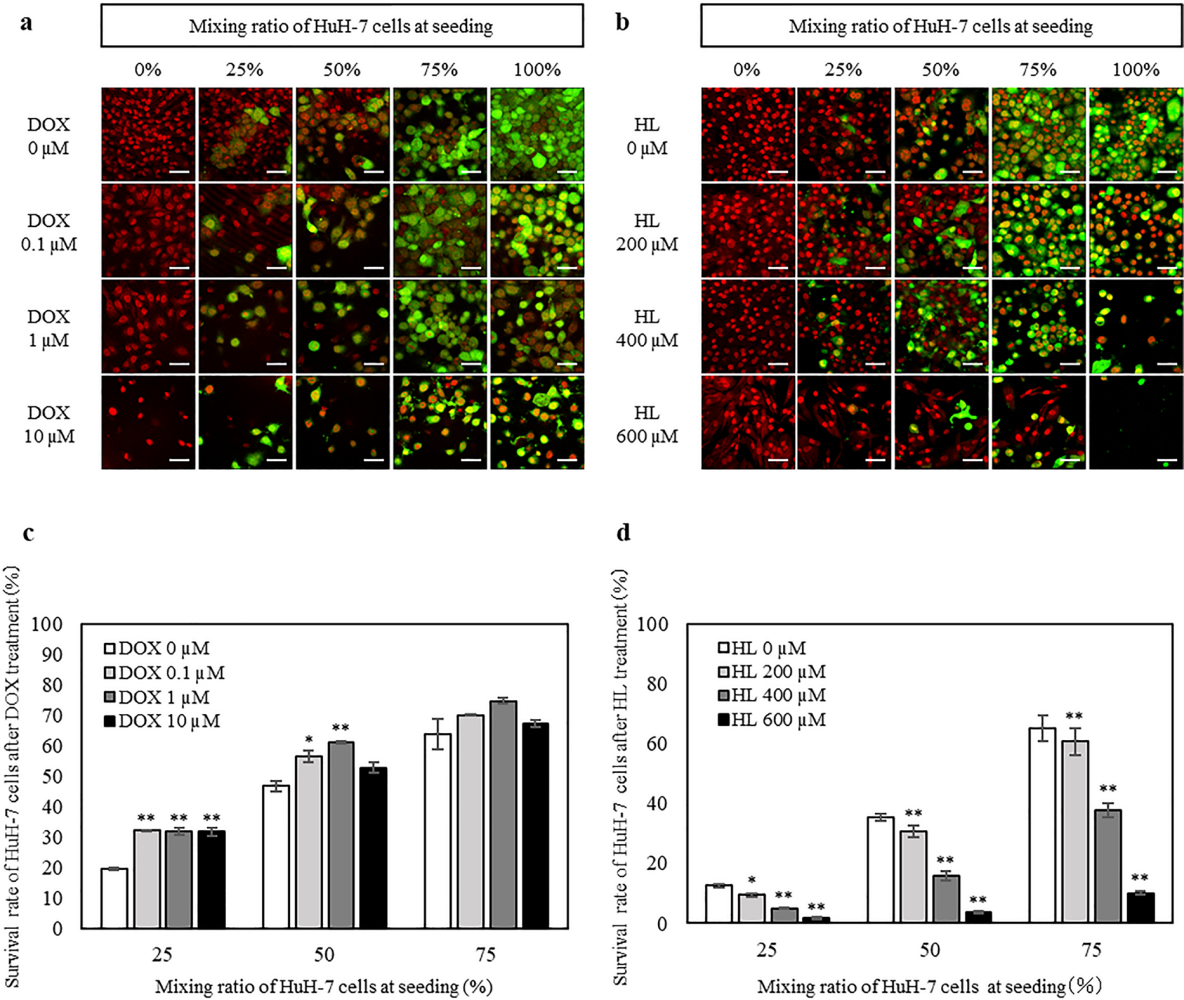
Changes in the ratio of remaining cells in a mixed culture of Hc and HuH-7 cells after DOX or HL treatment. HuH-7 cells were labeled with green dye and mixed with unlabeled Hc such that the percentage of HuH-7 cells to total cells was 0, 25, 50, 75, and 100%. **a** Confocal laser micrographs of the mixed cells treated with DOX for 48 h. Green indicates HuH-7 cells, and red indicates the nuclei of all cells. Scale bar represents 50 µm. **b** Confocal laser micrographs of the mixed cells treated with HL for 48 h. Green indicates HuH-7 cells, and red indicates the nuclei of all cells. Scale bar represents 50 µm. **c** Flow cytometric analysis of mixed cells treated with DOX for 48 h. Data represent the mean ± SE (n = 4). **d** Flow cytometric analysis of mixed cells treated with HL for 48 h. Data represent the mean ± SE (n = 4). *Indicates significant difference (*p* < 0.05) compared to the control (DOX 0 µM, HL 0 µM). **Indicates significant difference (*p* < 0.01) compared to the control (DOX 0 µM, HL 0 µM)

HuH-7 cells were tracked by dye-labeling only HuH-7 cells using a Green Cell Tracker, and all nuclei of the cells were stained with TO-PRO-3 (Fig. 5). Non-dye-labeled Hc cells and green-dye labeled HuH-7 cells were co-cultured (Table 1), the mixed cells were treated with DOX or HL for 48 h, and the cells were observed under a confocal laser microscope. Based on the results of the cell growth inhibition assay (Fig. 4), the exposure concentration of DOX was set to 0, 0.1, 1, and 10 µM based on the IC_20_ to IC_80_ values of HuH-7 cells and the IC_20_ value of Hc cells, while the exposure concentration of HL was set to 0, 200, 400, and 600 µM based on the IC_20_ to IC_80_ values of HuH-7 cells and the IC_20_ value of Hc cells.

DOX reduced viability and thus cell numbers under the 100% condition in only HuH-7 cells and under the 0% condition in only Hc cells (Fig. 5a). In contrast, HL reduced viability and thus cell numbers under the 100% condition but not under the 0% condition (Fig. 5b). In addition, when the ratio of HuH-7 cells in the mixed cells was 25, 50, and 75%, HL specifically reduced the number of HuH-7 cells, while the number of mixed Hc cells remained unchanged (Fig. 5b).

Next, after treating mixed cells of dye-unlabeled Hc cells and green dye-labeled HuH-7 cells with DOX or HL, the percentage of surviving HuH-7 cells was quantitatively assessed using flow cytometry. The percentage of HuH-7 cells among the remaining cells after DOX treatment showed no change regardless of the DOX treatment concentration (Fig. 5c). In contrast, the ratio of HuH-7 cells to the remaining cells after HL treatment tended to decrease with increasing HL concentration (Fig. 5d). These results indicated that HL selectively eliminated HuH-7 cells in a concentration-dependent manner, without altering the viability of Hc cells in mixed cultures.

### HL suppresses the tumorigenicity of the cells in dose-dependent manner

Finally, a soft agar colony formation assay was performed to assess the tumorigenic potential of the cells remaining after HL treatment (0, 200, 400, or 600 µM) for 48 h (Fig. 5).

Colonies were not formed under the 0% condition, which indicated the presence of Hc cells only, but the number of colonies increased as the percentage of HuH-7 cells in the mixed cells increased. Under the 100% condition, 214 colonies were formed per well.

HuH-7 cells formed colonies in the soft agar medium, indicating that they exhibit anchorage-independent growth, which is a characteristic of tumorigenic cells. In contrast, Hc cells did not form colonies in the soft agar medium and exhibited anchorage-dependent proliferation, which is known to be a characteristic of normal cells. Therefore, tumorigenic cells capable of forming colonies in a soft agar medium are HuH-7 cells.

**Fig. 6:**
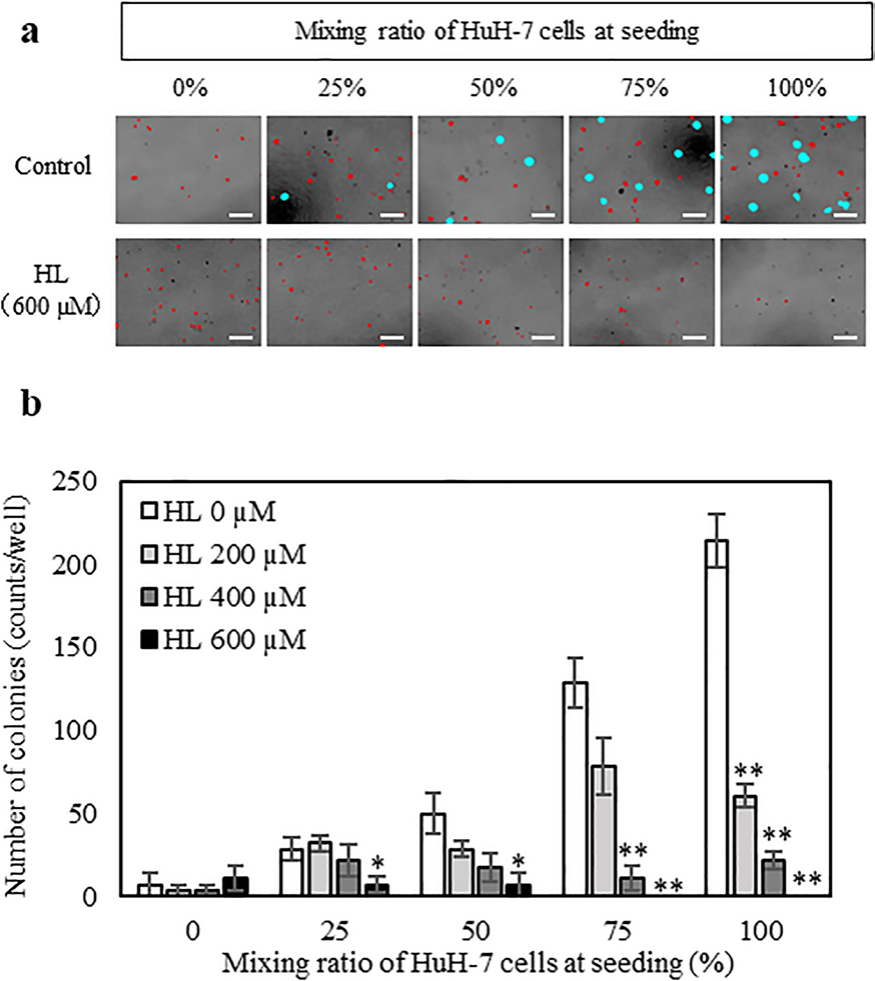
Soft agar colony formation assay of mixed Hc and HuH-7 cells remaining after HL treatment. **a** Photograph showing colony formation on a soft agar medium after 9 days. Colonies (shown in blue) were defined as areas larger than 5700 µm^2^ (Meyskens et al. 1984). Red color indicates cells that are viable but not undergoing aberrant proliferation (Meyskens et al. 1984). Scale bar represents 200 µm. **b** Changes in the number of colonies after HL treatment. Data represent the mean ± SE (n = 6). *Indicates a significant difference (*p* < 0.05) compared with the control. **Indicates significant difference (*p* < 0.01) compared to control

Colony formation in mixed cultures of Hc and HuH-7 cells was not confirmed under HL treatment (Fig. 6a). In addition, the number of colonies formed decreased in an HL concentration-dependent manner (Fig. 6b). These results suggest that HL treatment suppressed the tumorigenic potential of HuH-7 cells.

## Discussion

In this study, mixed cultures of normal human fetal hepatocytes (Hc cells) and hepatocellular carcinoma cells (HuH-7 cells) were used as normal and tumorigenic cell models, respectively, for a mixed tumorigenic cell population. We investigated the selective elimination of tumorigenic cells mixed with normal cells using HL. CS-C medium was used for the mixed culture of Hc and HuH-7 cells and it induced a high proliferation capacity in both cell types. The membrane fluidity of HuH-7 cells was found to be greater than that of Hc. Evaluation of the fusion and accumulation of HL in mixed Hc and HuH-7 cell culture showed that HL were more likely to fuse and accumulate in HuH-7 cells than in Hc cells. These results suggest that HL discriminate between normal and tumorigenic cells in the same environment, and selectively fuse and accumulate in tumorigenic cells.

The growth inhibition assay revealed that the inhibitory concentration range of HL was not overlapping between Hc and HuH-7 cells, suggesting that HL could selectively eliminate HuH-7 cells. Next, we evaluated whether HL could selectively eliminate HuH-7 cells in a mixed culture of Hc and HuH-7 cells by tracking HuH-7 cells in the mixed cells using Cell Tracker. HL selectively eliminated HuH-7 cells in a mixed culture while allowing Hc cells to remain viable. Finally, the tumorigenic potential of HL-treated cells was evaluated using a soft agar colony formation assay, which showed that HL suppress the colony-forming ability of HuH-7 cells.

These results indicate that in a mixture of normal and tumorigenic cells, HL selectively fuse and accumulate in tumorigenic cells and eliminate tumorigenic cells while allowing normal cells to survive in the same environment.

Previous studies (Nakano et al. 2002; Inamura et al. 2021) have evaluated the effects of HL on normal and tumorigenic cells in vitro in separate cell culture systems. However, experiments on the selective elimination of tumorigenic cells by HL in a mixed culture of multiple cells have not been conducted. In this study, we found that HL selectively fused and accumulated in tumorigenic cells, even in a mixture of normal and tumorigenic cells, and decrease the tumorigenicity of tumorigenic cells without affecting the viability of normal cells.

The ability of HL to selectively distinguish normal cells from tumorigenic cells can be attributed to their ability to distinguish between the membrane fluidity of normal and tumorigenic cells (Komizu et al. 2011a, b). Although the detailed mechanism remains unknown, the physicochemical properties of plasma membrane fluidity or substances that control plasma membrane fluidity are thought to be involved in the selective potential of HL. Elucidation of the precise mechanisms underlying HL activity warrants further research.

Regenerative medicine products intended for use in cell transplantation should have safety assurance regarding the existence of tumorigenic cells and should not adversely affect adequately differentiated cells that will serve as transplanted cells. In this regard, HL has been clinically administered intravenously in a total of 10 cases in 8 different cancers, including liver cancer. There have been no abnormal findings based on various hematological and biochemical tests, and the patients have achieved long-term survival of more than one year without side effects (Ichihara et al. 2008). Therefore, HL can be considered a safe reagent for introduction in vivo.

The results presented in this study are based on a mixed culture of normal human fetal hepatocytes (Hc cells) and hepatocellular carcinoma cells (HuH-7 cells) as a culture model for mixing a population of normal and tumorigenic cells for transplantation in regenerative medicine. The results of this study suggest that HL could safely eliminate tumorigenic cells, which are a risk factor for tumor formation in regenerative medicine, and support the application of HL to the quality control of regenerative medicine products. The use of HL for the quality control of regenerative medicine products could improve the safety of regenerative medicine products. In recent years, research using iPS cells has been actively conducted in regenerative medicine; hence, it is necessary to evaluate the emergence tumorigenic cells in a cell culture system using iPS cells. Since this paper demonstrated the efficacy of HL at the cell culture stage, we would like to investigate the exclusion of HL tumorigenic cells in an experimental system using iPS cells.

Contamination of tumorigenic cells can be a risk to the formation of tumors in vivo, even if a few cells are mixed in hundreds of millions of cells. However, many assays have detection limits when mimicking tumorigenic cell contamination in vitro. Therefore, means to detect contaminating tumorigenic cells have been explored in recent years, and there are increasing studies on sensitive methods to assess the presence of tumorigenic cells (Sato et al. 2019). In the present study, we examined the elimination of tumorigenic cells using HL in a mixed cell culture with a high percentage of tumorigenic cells; in the future, we will analyze the effect of HL in a culture containing a small percentage of tumorigenic cells.

In conclusion, the results of this study suggest that HL could selectively eliminate tumorigenic cells mixed into the cell population during the manufacturing of regenerative medicine products. Therefore, the application of HL promises to contribute to quality control for achieving safe regenerative medical products.

### Supplementary Information

Below is the link to the electronic supplementary material.

Supplementary file1 (PPTX 103 KB)

## Data Availability

The data underlying this study are available from the article and the corresponding author upon request.
