# Supplementary material for: Selective elimination of tumorigenic cells from mixed culture of normal and tumorigenic cells using hybrid liposomes aimed at realizing of cell therapy
Source: Cytotechnology. 2024 Feb 13;76(2):247–58. doi: 10.1007/s10616-023-00613-y (PMC10940552; doi:10.1007/s10616-023-00613-y)
Supplement: Supplementary file 1 — Supplementary file1 (PPTX 103 KB) [file 10616_2023_613_MOESM1_ESM.pptx]

## Slide 1
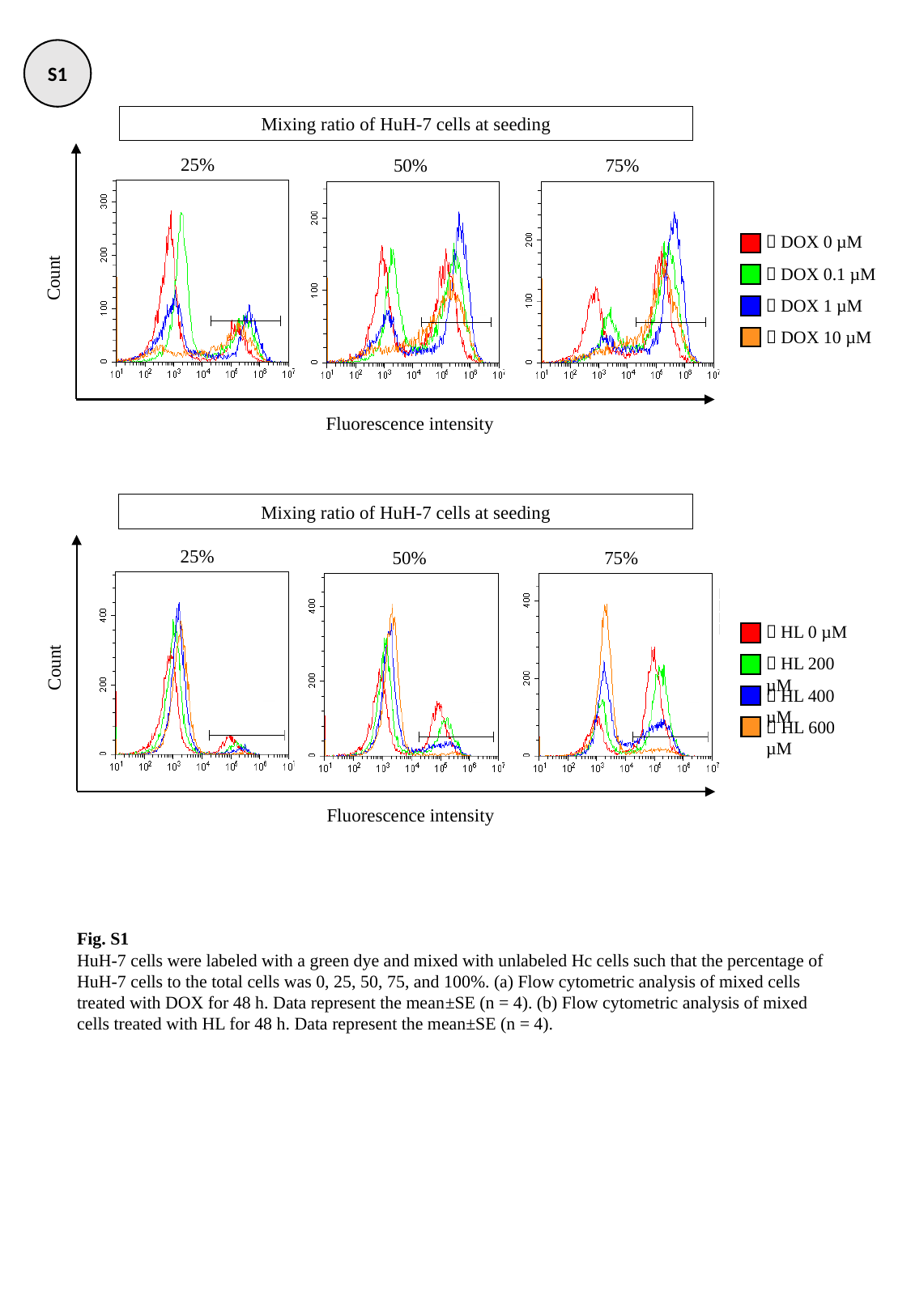

S1
Mixing ratio of HuH-7 cells at seeding
25%
50%
75%
Count
Fluorescence intensity
：DOX 0 µM
：DOX 0.1 µM
：DOX 1 µM
：DOX 10 µM
Mixing ratio of HuH-7 cells at seeding
25%
50%
75%
Count
Fluorescence intensity
：HL 0 µM
：HL 200 µM
：HL 400 µM
：HL 600 µM
Fig. S1
HuH-7 cells were labeled with a green dye and mixed with unlabeled Hc cells such that the percentage of HuH-7 cells to the total cells was 0, 25, 50, 75, and 100%. (a) Flow cytometric analysis of mixed cells treated with DOX for 48 h. Data represent the mean±SE (n = 4). (b) Flow cytometric analysis of mixed cells treated with HL for 48 h. Data represent the mean±SE (n = 4).
